# “*The vaccination is positive; I don’t think it’s the panacea*”: A qualitative study on COVID-19 vaccine attitudes among ethnically diverse healthcare workers in the United Kingdom

**DOI:** 10.1371/journal.pone.0273687

**Published:** 2022-09-09

**Authors:** Mayuri Gogoi, Fatimah Wobi, Irtiza Qureshi, Amani Al-Oraibi, Osama Hassan, Jonathan Chaloner, Laura B. Nellums, Manish Pareek, on behalf of the UK-REACH Collaborative Group

**Affiliations:** 1 Department of Respiratory Sciences, University of Leicester, Leicester, United Kingdom; 2 Lifespan and Population Health, School of Medicine, University of Nottingham, Nottingham, United Kingdom; 3 Department of Infection and HIV Medicine, University Hospitals of Leicester NHS Trust, Leicester, United Kingdom; Newcastle University, UNITED KINGDOM

## Abstract

**Background:**

Globally, healthcare workers (HCWs) were prioritised for receiving vaccinations against the coronavirus disease-2019 (COVID-19). Previous research has shown disparities in COVID-19 vaccination uptake among HCWs based on ethnicity, job role, sex, age, and deprivation. However, vaccine attitudes underpinning these variations and factors influencing these attitudes are yet to be fully explored.

**Methods:**

We conducted a qualitative study with 164 HCWs from different ethnicities, sexes, job roles, migration statuses, and regions in the United Kingdom (UK). Interviews and focus groups were conducted online or telephonically, and recorded with participants’ permission. Recordings were transcribed and a two-pronged analytical approach was adopted: content analysis for categorising vaccine attitudes and thematic analysis for identifying factors influencing vaccine attitudes.

**Findings:**

We identified four different COVID-19 vaccine attitudes among HCWs: Active Acceptance, Passive Acceptance, Passive Decline, and Active Decline. Content analysis of the transcripts showed that HCWs from ethnic minority communities and female HCWs were more likely to either decline (actively/passively) or passively accept vaccination—reflecting hesitancy. Factors influencing these attitudes included: trust; risk perception; social influences; access and equity; considerations about the future.

**Interpretation:**

Our data show that attitudes towards COVID-19 vaccine are diverse, and elements of hesitancy may persist even after uptake. This has implications for the sustainability of the COVID-19 vaccine programme, particularly as new components (for example boosters) are being offered. We also found that vaccine attitudes differed by ethnicity, sex and job role, which calls for an intersectional and dynamic approach for improving vaccine uptake among HCWs. Trust, risk perception, social influences, access and equity and future considerations all influence vaccine attitudes and have a bearing on HCWs’ decision about accepting or declining the COVID-19 vaccine. Based on our findings, we recommend building trust, addressing structural inequities and, designing inclusive and accessible information to address hesitancy.

## Introduction

Across the world, healthcare workers (HCWs) have been designated as a priority group to receive the COVID-19 vaccine due to their increased risk of exposure and transmission [1]. Uptake of vaccines by HCWs, however, has been influenced by vaccine hesitancy [2–7]. In the United Kingdom (UK), although overall vaccination rates among HCWs are high, recent figures published by the English National Health Service (NHS) show that acceptance of the COVID-19 vaccine is not uniform across age-groups, hospital Trusts, sexes, and ethnicities [8]. For example, data collected until the end of November 2021 showed that the proportion of Black Caribbean HCWs who had received two doses of the vaccine was 62.3%, as compared to 93.9% among White British HCWs during the same period [8]. Similarly, as of Feb 2022, cumulative vaccination rates (3 or more doses) among NHS Trust HCWs who appear in the NHS Electronic Staff Record (ESR), show that coverage among staff in the NHS London region (69%) is considerably lower than coverage in regions like the Northeast and Yorkshire (81.3%) and the South-West (84.1%) [9]. These differences gain further significance considering that in 2018 nearly 45% of staff in the NHS London region were from ethnic minority as compared to just 10.5% in the Northeast and Yorkshire region and 9.3% in the South-West region [10]. Large scale cross-sectional studies (including our own), conducted early in the roll-out of the COVID-19 vaccine programme in UK have also reported hesitancy among HCWs by ethnicity, gender and, job role [11–15].

Vaccine hesitancy has been defined by the World Health Organization (WHO) as “delay in acceptance or refusal of safe vaccines despite availability of vaccine services” and considers vaccine attitudes and behaviours to be part of a continuum that varies depending on time, place, and vaccine [16]. Another significant and early classification of vaccine attitudes was presented by Mark Nichter, an American anthropologist, who distinguished between *active demand* (adherence by an informed public) and *passive acceptance* (compliance to recommendations and social pressure) while looking at vaccine demand in low- and middle-income countries [17]. Nichter’s description of ‘*passive acceptance*’ particularly resonates in the current climate around COVID-19 vaccination where social norms or social influences are informing vaccine uptake [18]. As Yakub et al. [19] remarked, high rates of vaccine coverage often mask those who have hesitancy but accept vaccinations, but if this hesitancy is not understood and addressed in a timely way, the effectiveness and sustainability of vaccination programmes could be jeopardised.

Despite the early identification of vaccine hesitancy among HCWs in the UK, particularly among ethnic minority HCWs, it is concerning that hesitancy has persisted and not been addressed fully. Therefore, a rigorous exploration of HCWs’ views on COVID-19 vaccines and factors which influence these views is essential. This is critical to understand the uptake of COVID-19 vaccine booster doses and also to determine how policies such as mandatory vaccination could influence vaccine decision-making [20, 21]. In order to address these key questions, we carried out a qualitative study to investigate (a) HCWs attitudes towards the COVID-19 vaccine; (b) their perceptions about the vaccine; and (c) factors influencing vaccine decision making.

## Methods

### Study design, setting, and population

This qualitative study is part of a larger project, the United Kingdom Research study into Ethnicity And COVID-19 outcomes among Healthcare workers (UK-REACH), an Urgent Public Health (UPH) study to generate rapid evidence on COVID-19 outcomes among HCWs from diverse ethnic backgrounds in the UK [22]. We included adult HCWs (≥16 years of age) from clinical or ancillary roles and having experience of working in healthcare settings during the pandemic. HCWs from different ethnicities, sex, job roles, migration statuses, and UK regions were purposively recruited to obtain a diverse sample. We had a relatively large sample for a qualitative study but the size was determined by considerations of having adequate information power in our sample to address the study objectives and achieve analytical rigour [23]. Due to restrictions on travel and social distancing, the study was conducted remotely, and all processes including, recruitment, consent, and data collection were completed online. The study received ethical approval from the London-Brighton & Sussex Research Ethics Committee of the Health Research Authority (Ref No 20/HRA/4718).

### Recruitment and data collection

Data collection for this study was carried out between December 2020 and July 2021. We recruited participants through email invitation (containing the registration link) and advertising sent out via our collaborators and partner organisations to their member and staff mailing lists. Some of these organisations include the General Medical Council (GMC), General Optical Council (GOC), Health and Care Professions Council (HCPC), British Association of Physicians of Indian Origin (BAPIO), Filipino Nurses Association, UK (FNAUK), SERCO and more. We also conducted targeted recruitment through some of these organisations to ensure that we had representation from across job roles and UK regions. The national presence and coverage of these organisations meant that we received participants from across the seven NHS English regions (East of England, London, Midlands, North East and Yorkshire, North West, South East, South West, as well as the other devolved UK nations (Scotland, Wales and Northern Ireland). The study was also advertised on Twitter.

Prospective participants registered online, and were provided with a digital copy of the Participant Information Sheet (PIS) detailing the study, voluntary nature of participation and assurances of anonymity and confidentiality. Interested participants provided consent online, and filled in a short online demographic questionnaire, which included information on age, sex, ethnicity, job role, job location, and country of birth. Recruitment was then guided using purposive sampling. Potential participants were invited to take part in either a semi-structured interview or focus group, depending on their choice. Our choice of using both focus groups and individual interviews for data collection was based upon the understanding that semi-structured interviews would allow us to identify or explore in detail personal experiences and insights that were important to participants [24]. Additionally, we choose interviews because we felt that sharing of sensitive information such as experiences of discrimination may be easier for participants in an interview setting. Alternately we choose focus group to explore HCWs experiences and opinions in a shared environment which would bring out the similarities and differences in experiences and provide greater breadth to the data [24].

A topic guide was developed in consultation with the UK-REACH Professional Expert Panel (PEP) and stakeholder group (STAG), the public engagement and stakeholder engagement groups respectively. The topic guide explored HCWs experiences of working during the pandemic, their fears, concerns and perceived risks, challenges encountered, support and facilitating factors etc. It also contained a section on vaccine where the participants were asked questions about whether they had been offered or had a COVID-19 vaccine; vaccine intentions; reasons behind vaccine decisions and; thoughts on the safety, access and delivery of the vaccine (See S1 File). The topic guide was piloted with the first eight participants, and refined iteratively during data collection where new key issues emerged (e.g. changes in vaccination policies) to ensure it was relevant and current. Interviews lasted for 45-60mins and focus groups took about 1.5 hours, with group sizes varying between 2 to 7 members. Following their participation, a gift voucher was given to HCWs in recognition of their contribution to the research.

Interviews and focus groups were conducted either through Microsoft Teams or by telephone (only interviews) by MG, FW, AAO, LBN, OH, and IQ who are all trained qualitative researchers and have prior experience of working with culturally and ethnically diverse communities. Interviews and focus groups were recorded with prior permission of the participants using the recording feature in Microsoft Teams or using an encrypted recorder during the telephone interviews. Recordings were anonymised and transcribed by professional transcribers, and checked for accuracy by the research team.

### Data analysis

We adopted a two-pronged approach to analyse the transcripts. We conducted a content analysis [25] of all the transcripts to inform our categorisation of vaccine attitudes for all the participants. Two researchers (MG & JC) looked at all the transcripts and identified the sections where participants had spoken about vaccination and coded these individually into one of the attitude categories that were developed through analysis of the first 20 transcripts. These individual coding were tallied (agreement was >80%), and differences resolved after thorough discussions. Simultaneously, we also undertook thematic analysis using a data-driven inductive coding approach [26] to identify the factors influencing vaccine decision-making among HCWs. One of the researchers (MG) developed the coding framework and generated the first set of themes based on coding a subset of transcripts on NVivo. These were then shared with the team, and all the researchers then iteratively developed the framework, as they looked at the wider set of transcripts. Regular weekly meetings helped the team deliberate upon any new themes that emerged and revising the coding framework as we continued coding the transcripts. The initial set of themes was also presented to the PEP and STAG and revisions made on the basis of the inputs received from the members. The final themes were arrived at after no new themes emerged from the iterations and all the team members were in agreement with the generated codes and themes.

## Results

### Demographic data

We recruited 164 participants from December 2020 until the end of July 2021, and conducted 103 interviews and 16 focus groups with these participants. Demographic details of participants are provided in Table 1. To clarify, one participant who identified as ‘Other’ was randomly assigned to the Female sex category to limit identification.

**Table 1:** Demographic characteristics of participants.

Variable	N (%)
**Sex**	
Male	63 (38.4%)
Female	101 (61.6%)
**Age, median (IQR)**	42 (32–53)
**Ethnicity**	
Asian	65 (39.6%)
Black	29 (17.7%)
Mixed	15 (9%)
White^a^	49 (30%)
Other	6 (3.7%)
**Job Role**	
Doctors	44 (26.8%)
Nurses & Midwives	30 (18.3%)
Allied Health Professionals^b^ (AHP)	62 (37.8%)
Ancillary Health Workers^c^ (AHW)	28 (17.1%)
**Country of Birth**	
UK	92 (56.1%)
Outside UK	70 (42.7%)
Missing	2 (1.2%)
**Years lived in the UK (for those born outside UK)**	
0-2yrs	4 (5.7%)
2-5yrs	5 (7.2%)
5-10yrs	10 (14.3%)
>10yrs	50 (71.4%)
Missing	1 (1.4%)
**UK Region**	
England	140 (85.4%)
Scotland	7 (4.3%)
Wales	3 (1.8%)
Northern Ireland	10 (6.1%)
Unknown	4 (2.4%)

^a^Includes White British, White Irish, White Gypsy/Traveller and White Other

^b^Also includes dentists, pharmacists, healthcare scientists, ambulance workers and those in optical roles.

^c^Includes those in administrative, or other non-clinical roles (e.g. housekeeping/security/maintenance etc.)

### COVID-19 vaccine attitudes

HCWs participating in the UK-REACH qualitative study demonstrated a range of attitudes towards the COVID-19 vaccine, which could be arranged into four distinct categories: Active Acceptance, Passive Acceptance, Passive Decline, and Active Decline. The categories have been inspired by Nichter’s ‘*active demand*’ and ‘*passive acceptance*’ classification, and been extended to include two more categories to cover those attitudes which fall towards the non-acceptance end of the spectrum. Considering the scepticism or doubt about the vaccine present in all three categories of Passive Acceptance, Passive Decline, and Active Decline, these have be regarded as vaccine hesitant. Table 2 provides a description of the four vaccine attitudes, with some representative quotes.

**Table 2:** COVID-19 vaccine attitudes among HCWs.

Attitude Category	Description	Representative Quote(s)
Active Acceptance (AA)	Participants who demonstrated ’active acceptance’ were those who readily accepted the COVID-19 vaccine, and reported that they were vaccinated or willing to be vaccinated when offered.	*About the vaccine…there is nothing to be scared about this vaccine*. *These scientists have done a very*, *very good job to get us the vaccine*. *Please*, *everyone should go for it*. *There’s nothing*, *nothing to be scared about*. *I will assure everybody to go for their vaccine*.*”* (P18, AHW, Black)
		*I think it’s imperative that we’re all encouraged to get a vaccine*. *Personally*, *I’d be very keen to have a vaccine at the opportunity that it’s afforded to me; my wife’s the same*.” (P4, Doctor, Asian)
Passive Acceptance (PA)	Participants who demonstrated ‘passive acceptance’ often had one dose or both doses of the vaccine, but had concerns around its safety or efficacy. Participants said that they were initially fearful of the after-effects of taking the vaccine or its long-term effects, but decided to get the vaccine for a variety of reasons.	*Obviously*, *initially I was a bit hesitant about the vaccine because I think*, *like the majority of people*, *it just felt like it came out really fast*, *and knowing how vaccines and medicines are*, *it can take years for them to get approved*.*”* (P55, Nurse/Midwife, Black)
		*I’ve had my first one and my second one’s tomorrow*. *I mean*, *I’m really pleased and I’m really grateful to be getting it*, *but then you watch on the news and they say about ‘Oh there’s these other variants and the vaccine’s not as strong*,*’ then you start thinking ‘oh no*, *just when you thought you were seeing light at the end of the tunnel’… and you think oh is it going to ever end*?*”* (P63, AHW, White)
Passive Decline (PD)	The ‘passive decline’ category, which includes those who have been offered a vaccine or have access to it, but are delaying having the vaccine and remain indecisive if they would take the vaccine or not.	*I have allergies…I do worry about having the vaccine when I seem to have all*, *exacerbated my allergies…I do have concerns…my* [family member] *has had* [adverse reactions] *following some previous immunisation…and with my allergies* [and health conditions], *I am fearful that I might end up having* [similar adverse reactions]…*and so yeah*, *so I have got some fears about vaccination*.*”* (P76, Nurse/Midwife, Mixed).
Active Decline (AD)	Participants in this category are those who said that they had not taken the vaccine when offered or did not intend to take the vaccine.	*I’m not taking vaccine because*, *you know*, *the second wave I didn’t got COVID*, *I’ve got my natural immunisations*, *so I’m not really fancying the vaccine either*.*”* (P40, AHP, White)
		*I was offered* [at work]*…But I declined…I had concerns it is developed too quick*, *not proper testing is done or it’s not sufficient*.*”* (P16, AHW, Asian)

Our data show that out of 164 participants, 91 (55.5%) had actively accepted the vaccine, 42 (25.6%) had passively accepted, and six (3.7%) each had passively or actively declined the vaccine. Vaccine attitudes could not be clearly ascertained for 19 (11.5%) participants and hence have not been categorised. We found that out of the 54 participants who had passively accepted or declined (passively or actively) the vaccine, most (n = 40, 74%) were female. Moreover, majority of participants who shared hesitant views were from ethnicities other than White British (n = 47, 87%). In addition, all 12 participants who had declined (actively or passively) the vaccine were from ethnic minority groups, with one third (n = 4) of them identifying as Black. Most participants (n = 20, 48%) who had accepted the vaccine passively were of Asian ethnicity. Additionally, more than half (n = 28) of those who had passively accepted or passively/actively declined the vaccine were from allied health professions or working in ancillary roles.

### Factors influencing COVID-19 vaccine attitudes

There were several factors that were found to influence acceptance of the COVID-19 vaccine among HCWs. These can be grouped under five broad categories: trust; risk perception; social influences; access and equity; and considerations about the future.

#### Trust

Trust was central in shaping participants’ attitudes towards the COVID-19 vaccine. Our analysis found that HCWs weighed trust at two inter-linked levels: trust in vaccine and trust in governance. In both cases, lower trust has been found to influence lower uptake of the vaccine and vice-versa. Also, those who mistrusted both the vaccine and the governance structures were more likely to passively or actively decline the vaccine.

*Trust in vaccine*. Trust in the vaccine was shaped by participants’ knowledge of vaccine science. Those who demonstrated familiarity with medical, scientific, or public health information about vaccines were mostly from clinical backgrounds and they were also the ones who were most confident about the COVID-19 vaccine and actively accepted it. Some of these participants had acquired knowledge of vaccinology and virology as part of their professional curriculum or training, and some others had acquired this knowledge during the course of their job. This knowledge seemed to have boosted participants’ confidence around the COVID-19 vaccine and led to uptake.

*“Yes*, *I was very*, *very*, *very optimistic*, *because A*, *there was some research*, *even though it was speedy*, *but it was necessary at the time*, *and that was the best that they could come up with*. *Although it’s an RNA virus*, *which of course we know the modus operandi of RNA virus as against DNA viruses*, *the fact that the vaccine can affect an RNA virus is a little bit safer for humans in my little understanding of virology*, *than DNA viruses*, *vaccines*, *which can then*, *of course*, *cause re-combinations with our own DNA*. *But we don’t have DNAs*, *fortunately*. *So*, *if something is able to protect us against an RNA*, *it’s quite*, *it’s less likely to cause mutation in our own DNA and cause us any harm as compared with the vaccine supposedly fighting a DNA virus*. *So*, *I’m very optimistic about it*.*”* (P53, Doctor, Black, AA)

Confidence in vaccines, generally, was also an important factor influencing participants’ trust in the COVID-19 vaccine. Participants who accepted the COVID-19 vaccine, either actively or passively, discussed their uptake of other recommended vaccinations both for themselves and their families. The association between flu vaccine and the COVID-19 vaccine was made by several accepting participants. One participant who demonstrated ‘active acceptance’ said:

*“I wasn’t hugely concerned about it really*. *I get the flu jab every year*. *I’ve had other vaccines in the past*. *I’m a believer in vaccination programmes*.*”* (P89, Nurse/Midwife, White, AA)

As opposed to those stating higher trust in the COVID-19 vaccine, several other participants expressed their lack of trust in the vaccine and had therefore declined it or accepted it with reservations. Such participants described a key cause of doubt about the vaccine was the pace at which it was developed.

*“If I’m honest*, *I was concerned*, *because of the fact that they’d produced a vaccine so quickly*.*”* (P66, Nurse/Midwife, Asian, PA)

Related to the concerns about the expedited vaccine development process, participants also specifically talked about their apprehensions regarding the lack of long-term data to rule out any unknown side-effects. For many female participants, the unknown effect of the vaccine on fertility had been singularly important in creating a mistrust about the vaccine and influencing their vaccine decision. As one participant remarked:

*“I have had my first vaccine*, *albeit slightly delayed*, *I definitely did question whether I was comfortable to have the vaccine*, *not from a race perspective so much as from a fertility perspective I was concerned*.*”* (P83, AHP, Black, PA)

Participants also questioned the effectiveness of the vaccine itself, and were wary that with new variants emerging, the vaccine might not be sufficient to see an end to the pandemic, which may have resulted in residual hesitancy.

*“So*, *for me*, *the vaccination is positive; I don’t think it’s the panacea*. *It’s a Coronavirus*, *it mutates*, *we’ve already seen that*, *they’re calling it a variant–it’s mutated basically… this was going to happen…so I don’t know*…*how much propaganda is in this*, *maybe it is true*, *maybe the vaccine that they’ve developed will protect us or maybe they’re behind the curve again and they’ve got to redesign the vaccine…”* (P2, AHW, Asian, PA)

For some ethnic minority HCWs, the concern that the vaccine had not been adequately tested on people of colour made them hesitant.

*“I was very apprehensive initially about taking the vaccine at this early stage because of the history of tests that were carried out on our ethnic group and just not knowing whether or not there was going to be any side effects to this vaccine*.*”* (P87, AHP, Black, PA)

*Trust in governance*. Trust in the vaccine was closely linked to trust in institutions producing or delivering the vaccine such as pharmaceutical companies, the government or even the NHS.

*“I think my concerns about the vaccine are principally linked to…when the government etcetera imposed a lot of responses which from my point of view are patently disproportionate*, *it’s difficult to then trust them on any other kind of things they might suggest to tackle it*.*”* (P84, Doctor, Mixed, AD)

Past clinical malpractices or history of discrimination and racism against ethnic minority communities have created distrust of the COVID-19 vaccination campaign. For example, several of our participants spoke about the drug trial carried out by Pfizer in Nigerian children in 1996 without proper ethical clearance [27], and how such instances remain in the collective memory of the community and can make people distrustful of vaccines, particularly the newly developed ones like for COVID-19. Some participants also spoke of historical discrimination and racism and how it has made people from ethnic minority communities question the credibility of the intention to vaccinate NHS staff. Racism in the NHS is a long-drawn problem and workplace surveys have reported staff from ethnic minority communities being subject to bullying, discrimination and harassment at work, as well as being side-lined for career progression [28]. As one participant who had accepted the vaccine but spoke of the sentiments among ethnic minority communities

*“There has been a lot of mistrust among the community*, *for example some of the communities will be asking why suddenly is NHS interested in the Black and Asian minority ethnic group*? *Over years we have spoken*, *they’ve never listened to us*, *what has changed now*, *what is the intention here*?*”* (P59, Doctor, Black, AA)

In the words of another participant:

*“I know it’s good for me*, *however*, *why is it now just the BAME people and the vaccine*? *Why wasn’t we given PPE*, *then*? *Why wasn’t we given vitamin D*, *then*, *to prevent us*? *Why did we take a back seat*, *then*, *if it predominantly affected us*? *All these questions are unanswered*, *yes*?*”* (P39, AHP, Black, AD)

#### Risk perception

We found that participants in our study made their decision about vaccination based on perceived risks, and those who considered the risk of infection or severe disease to be high indicated a greater acceptance of the vaccine, both actively and passively. Participants considered a range of factors in their perception of risk to self, family and even their patients, which in turn influenced their decision to get vaccinated. For example, some participants expressed concern about the risk of exposure in their role but, at the same time considered themselves to be at low risk of getting severely ill if they were infected, so accepted the vaccine as a precautionary measure. As one actively accepting participant shared:

*“I suppose myself I think I had sort of*, *like*, *few of the risk factors personally… so I don’t think that I’ve ever felt particularly that I was at risk of becoming detrimentally unwell for my own health…*[but] *the fear of the unknown I suppose and I guess you’re putting your own health at risk*, *you’re putting that of anybody that you live with at risk*, *I was always scared about going to work and catching the virus and then passing it on…”* (P8, Doctor, White, AA)

Another participant spoke of her vaccine decision:

*“Initially I wasn’t sure about the vaccine*, *if I was going to have the vaccine …The reason I had the vaccine again is because looking at the evidence that I believed and the people that I believed showed that by having it*, *even if I was to get the coronavirus*, *that hopefully I wouldn’t get the severe form and that would sort of hopefully reduce my risk of ending up in hospital or seriously ill*.*”* (P90, Doctor, Black, PA)

Whilst risk to self was an important consideration for many participants, risk to others was often a more significant concern. Participants shared being fearful or worried about infecting their families and loved ones, and even patients, and these concerns led them to accept the COVID-19 vaccine, either actively or passively. As is evident from what one participant said:

*“The reason I did it was one*, *not to protect myself*. *I’ve never been scared of anything…But I took the vaccine really to*, *for my mum really*, *because I was working in a higher risk environment*.*”* (P77, AHP, Asian, PA)

However, it’s important to note that if the risk of disease is deemed to be low, vaccine acceptance may be adversely affected.

*“We’re all young so we can take that risk really*. *So I’ll just leave it up to the older people to have it and then I’ll just see what effects it has on them first*, *pretty much*.*”* (P91, AHP, Black, AD)

#### Social influences

Amongst our participants, social influences, or what others in their social group were doing and saying about the vaccine, seemed to have considerable impact on HCWs’ decision to get vaccinated. As one participant, who had concerns about the safety of the vaccine but took it after being reassured by her friend, said:

*“I have a family member who works in clinical trials who said that the whole process had been fast tracked and he was of the view that it was absolutely completely safe…his view overall was that it was definitely better to have it than not*, *and I did take it*.*”* (P66, Nurse/Midwife, Asian, PA)

On the flip side, while the influence of friends and family had facilitated most participants in accepting the vaccine, for a few it had actually resulted in creating doubts and apprehensions about safety.

*“It’s only because you’re hearing this all side effects of vaccines*. *That was a bit worry … I heard from one of my friends who works in a different hospital*, *one of the people had the vaccine and they collapsed in less than 3 minutes*.*”* (P85, Nurse/Midwife, Asian, PA)

While for some participants, opinion of others in their immediate social network influenced their decisions about the vaccine, some other participants influenced their close family and friends to get vaccinated. A nurse, who has family living abroad said:

*“I recommended my parents who live in* [Place X], *my parents in their late 80s*, *I said to them*, *‘When the vaccine come in*, *starting*, *you go and take your vaccination to protect yourself and others*.*”* (P7, Nurse/Midwife, Asian, AA)

Certain other participants also made endorsements through social and/or popular media to influence members of their community to get vaccinated.

*“I’ve done a lot of* [media] *interviews*, *personally…just to try and get the message out that to take this Covid-19 seriously…”* (P56, AHP, Asian, AA).

These quotes reflect how social influence work both ways and while HCWs can get influenced by family, friends or colleagues to accept or decline the vaccine they in turn can also play a role in determining public attitudes towards vaccination. Some participants also shared about the expectation from HCWs to support and promote vaccination which at times may result in unaddressed fears and concerns and thereby increase hesitancy:

*“I know that even amongst some of my colleagues there is still a reluctance to take the vaccine which sort of beggars belief considering that they are all medical people who I would have thought have understanding of the sciences but well*, *everyone’s different*.*”* (P48, Doctor, Asian, AA)

#### Access and equity

Another category of factors which we found had an influence on participants’ decision making about the COVID-19 vaccine was related to access and equity, which are important measures of ‘vaccine convenience’ that could trigger hesitancy [16]. Many participants expressed how inequities in vaccine delivery and access, information dissemination and messaging, and official decision-making about the vaccine had affected or can affect vaccine uptake not just among HCWs but also within the wider community.

*Vaccine access and equity*. While accessing vaccines was straightforward for many participants, some felt that working on temporary or locum contracts made access challenging. One participant, who is a trainee doctor, shared:

*“There was a little concern about some of us*, *who work in locum capacity*, *because obviously*, *if you are a locum doctor you are not tied to anyone*, *so just*, *you’re like a mercenary soldier*, *you see*? *You are*, *so nobody has any direct responsibility for you*, *unless your GP*, *and your GP cannot do that*.*”* (P53, Doctor, Black, AA)

Some participants also expressed their dissatisfaction with the way delivery of vaccines were prioritised, and questioned why certain groups such as nurses, ethnic minority staff, and community-based professionals such as podiatrists were not prioritised early on despite close contact with patients or having known to be at greater risk. As one participant remarked:

*“And actually what I felt was*, *which was very wrong and I’ve expressed it*, *is that we were doing all these jobs without the vaccine*, *OK*. *So people that weren’t working in intensive care they’ve already been vaccinated and I didn’t feel that* [HCWs] *in intensive care were given priority and they should have been given priority for the vaccine*.*”* (P51, Nurse/Midwife, Asian, PA).

*Information access and equity*. Some participants rued the fact that they didn’t have easily comprehensible information or credible sources for consultation, which meant that they experienced confusion and panic while making vaccine decisions.

*“They can’t tell me whether I need to have–I can’t–they gave me my vaccine* [first dose] *and they can’t answer*, *like*, *I can’t find somebody to ask the question*, *questions I need answers for about my second vaccine*. *I can’t find the right person to talk to*.*”* (P86, AHP, Other, PA)

A lack of timely or accessible information was another issue which influenced participants’ confidence in and decision making around the vaccine.

*“I think what would have benefitted in my case was having the leaflet*, *the vaccination leaflet*, *before I had the vaccine rather than being given that after I’ve had it because it included a lot of information that people could benefit from*, *that I could have benefitted*, *and I didn’t have to go fact finding and I wouldn’t have had that doubt if I knew exactly what to expect and how it worked and what it was*.*”* (P58, AHP, Asian, PA)

Many ethnic minority participants also talked about the influence of misinformation or misleading information that gets circulated in the community, in the absence of adequate and accessible government messaging, and the need to address this in order to support vaccine delivery. In the words of a participant,

*“I think the government should be spending a lot more money and getting a lot more out there within the communities and making a big effort to give out the proper information…there’s communities within communities*, *and that’s who we have to tackle*, *because this where the*, *kind of*, *in the core*, *the misinformation starts and it just kind of starts spreading*, *you know*, *and so we’ve got to tackle it from the core*.*”* (P56, AHP, Asian, AA)

Participants also shared how the decision made in early 2020 to increase the gap between the two vaccine doses caused them anxiety. Most participants described that they were not given a justification about this change, which gave them an impression that the decision was more of “*a financial decision and not exactly science*” (P47, AHW, White, AA) and another “*shifting of goalposts*” (P8, Doctor, White, AA) by the government.

*Mandatory vaccination and equity*. *W*hen discussing their perceptions about the COVID-19 vaccine, some HCWs raised the issue of mandatory vaccination. Views around mandatory vaccination for HCWs or even vaccine passports were divided. There were some HCWs who felt strongly that vaccines should be made mandatory for healthcare staff, but others felt that it would impinge on people’s personal freedom and create further inequities among HCWs.

*“I also feel like if you do kind of penalise people for not being vaccinated*, *disproportionately the people who aren’t being vaccinated are the people who are already kind of maybe more vulnerable in their workplace or in their finances and kind of lose out twice–not being treated well by the government and then lose out from this*.*”* (P81, Doctor, Mixed, AA)

These sentiments along with preference for non-coercive strategies for improving vaccine uptake may have influenced HCWs vaccine decision and also their opposition to the UK government’s mandatory vaccination order [21], which has now been revoked following a public consultation in February 2022 [29].

*“It’s never been suggested before that everybody should be vaccinated for something of this sort…We don’t mandate the flu vaccine*. *Those are my reasons for myself not wanting it*. *I’m not opposed to people choosing to have it themselves if they want but I don’t like the idea that people should be vilified for not wanting it*.*”* (P84, Doctor, Mixed, AD)

#### Considerations about the future

One of the biggest drivers of COVID-19 vaccine uptake among our participants was the belief that the vaccine could be a way out of the pandemic. Participants, although hesitant about taking the vaccine, were driven to uptake by considerations such as, “*…try and get back to normal or as normal as we can be*” (P88, AHP, White, AA) and “*A step forward to freedom to a normal life*…” (P51, Nurse/Midwife, Asian, PA). The will to protect oneself for the sake of their family and children was also a consideration for certain participants. As one participant shared:

*“So*, *just weighing it up*, *I just thought*, *‘I’m better off having it*, *and then*, *if I do get COVID*, *it’ll hopefully… I’ll survive it and won’t have any long-term issues or end up six feet under*.*’ So*, *that was the biggest motivator*, *and I just thought*, *I need to be here for the kids*, *because they’re not*, *obviously*, *old enough to look after themselves*.*”* (P55, Nurse/Midwife, Black, PA)

For several other participants, the hope that vaccination would allow them to travel was a significant reason for getting vaccinated. One participant who has family living abroad said:

*“I mean*, *it’s probably going to go on and on but with the vaccine hopefully the impact on people’s lives will not be as much and so then I will be allowed to travel soon to see my parents”* (P61, AHP, Asian, AA).

## Discussion

Our study is the largest and one of the first qualitative studies with an ethnically diverse sample of HCWs across the UK to explore attitudes towards COVID-19 vaccination. HCWs were prioritised for receiving the COVID-19 vaccine, but there have been disparities in vaccine uptake in this population based on a host of factors. While these disparities have been identified, comprehensive understanding of vaccine attitudes underpinning these variations are yet to be fully explored. In this context, our study brings in-depth understanding of the spectrum of hesitancy among HCWs, their attitudes towards the COVID-19 vaccine, and the factors which are crucial in influencing vaccination decisions.

Our categorisation of vaccine attitudes offers a novel and comprehensive lens to examine the range of COVID-19 vaccine attitudes which HCWs display, and also how these attitudes could change with changing circumstances. In our study, most HCWs reported positive attitudes towards the COVID-19 vaccine and actively accepted it; but a significant number of HCWs also expressed reservations about the vaccine and had only accepted it passively. This category is important, as it highlights underlying hesitancy and shows that even people who have received both doses of the vaccine can remain sceptical about the efficacy and need for the vaccine. This underscores both that hesitancy is normative, and must be addressed rather than stigmatised or dismissed. Furthermore, if this scepticism is not addressed in a timely manner, there are chances that passively accepting individuals could move towards the declining end of the spectrum. This is especially critical now when booster doses have been initiated, alongside concerns that vaccination may not be effective against new variants.

The sizeable proportion of ethnic minority HCWs showing vaccine hesitancy is in line with previous studies [12, 14, 15, 30]. This finding highlights that ethnic inequalities and discrimination, predating the pandemic, have underpinned mistrust and hesitancy among ethnic minority HCWs towards the COVID-19 vaccine [30]. The lack of easily comprehensible information, and rapidly changing government guidelines such as increasing the gap between dosages, and even in other areas such as PPE use, may have further eroded the already fragile trust in authorities. Addressing vaccine hesitancy among ethnic minority HCWs, therefore, has to start with efforts at building trust through empathy, active engagement and attentive listening, and most of all, discarding pejorative attitudes towards these communities. This could start with culturally competent approaches coupled with meaningful inclusion and proactive efforts to eradicate systemic racism and structural discrimination to ensure ethnic minority staff are valued [31]. Without all of these, vaccine promotion strategies, like mandatory vaccination, may seem enforced, exacerbate power inequities, disempowerment, and marginalisation, and increase distrust [32]. In fact, coercive ways of improving vaccine uptake like mandatory vaccination may prove counter-productive as hesitancy may get further entrenched if HCWs felt pressured to get vaccinated against their free will [15]. Additionally, the higher rates of vaccine hesitancy among female HCWs indicate that gender issues such as fertility, pregnancy and personal care responsibilities (fears of vaccine side-effects impacting ability to care) had not been well anticipated before roll-out, which left a wide gap for conspiracy theories to flourish and fuel people’s fears. Moreover, the finding from our as well as other studies that acceptance of the COVID-19 vaccine differs by ethnicity, gender and job roles means that addressing vaccine hesitancy needs an intersectional approach that looks at all these factors in an integrated way [30].

Our findings also highlight that vaccine decision-making is a complex process for some HCWs as they consider several factors before deciding to take the vaccine, delay it, or decline to have it. Like other studies [33, 34], perception of risk to self and to others particularly, have been found to significantly influence vaccine decisions among our participants too. Furthermore, the role of social networks in influencing vaccine acceptance and uptake among HCWs is also significant [18]. In line with previous research, our study also found that inequities in accessing appropriate vaccine information could affect vaccine uptake [15, 30]. Furthermore, some of the considerations identified in our data, such as risk and exposure at work, duty of care and, mandatory vaccinations are unique to HCWs and have a bearing on their decision-making about the COVID-19 vaccine [30]. This complexity implies that measures to improve vaccination rates among HCWs have to be multi-pronged and dynamic to generate greater appeal and acceptance.

Our study has certain limitations. For example, social distancing measures meant that data collection and study promotion had to be conducted remotely and using online technology. This may have affected participation from certain groups who may be less proficient in use of or have less access to digital technology. Relatedly, this may have resulted in selection bias and there is a likelihood that more number of participants who have had the COVID-19 vaccine may have taken part. However, this study had a unique advantage of being able to collect data in real-time as the situation around COVID-19 vaccination has been unfolding in the UK, although the rapid change in scenario could also mean that some of the participants’ views may have changed from the time of data collection. Given this, we are making efforts to keep pace with the changes and bring out results rapidly, and some of our early results have already been considered by the government in their policy-making decisions [35] Additionally, while we collected substantial demographic data from participants, certain information such as education level of participants was not asked. However, we had a large cohort study conducted as part of the project and extensive details on level of education, years in employment etc. have been collected as part of that and could help explore links between vaccine decision-making and educational levels among HCWs [36].

## Conclusion

In conclusion, our study is one of the first to report in-depth qualitative data on attitudes of ethnically diverse HCWs towards the COVID-19 vaccine and factors influencing vaccination decisions. While vaccine hesitancy among HCWs has been reported by other studies, our research further delineates some of the groups’ susceptible to hesitancy and also brings out the factors behind this hesitancy, such as trust and risk perception. Based on our findings, we recommend that trust-building, inclusive and accessible information, and commitments to proactively addressing structural inequities are going to be crucial in improving vaccine uptake among HCWs.

## Supporting information

S1 File Topic guide areas. (DOCX)
