# Supplementary material for: “The vaccination is positive; I don’t think it’s the panacea”: A qualitative study on COVID-19 vaccine attitudes among ethnically diverse healthcare workers in the United Kingdom
Source: PLoS One. 2022 Sep 9;17(9):e0273687. doi: 10.1371/journal.pone.0273687 (PMC9462779; doi:10.1371/journal.pone.0273687)
Supplement: S1 File — (DOCX) [file pone.0273687.s001.docx]

**Supporting Information S1**

**Topic Guide Areas**

| **OBJECTIVES**   - To gather reflections on participants’ views and experiences as clinical and non-clinical healthcare workers during the COVID-19 pandemic; - To explore what factors healthcare staff feel have put them or other healthcare staff at risk; - To examine fears or concerns healthcare staff have experienced both in and outside of work; - To discuss challenges healthcare staff have experienced in accessing the information they need for protecting themselves; - To identify things that have been helpful in their workplace or community in supporting healthcare staff or protecting their health. |
| --- |

**INTRODUCTION**

- Introduction about researcher, project, and funder
- General informed consent information, e.g. how the data will be used, confidentiality, timing, permission to audio-record and transcribe interview/focus group

**BACKGROUND INFORMATION**

**Big Idea**: participant’s life experiences (within and outside of healthcare settings) may shape their perspectives on risk factors, challenges, fears, or helpful resources during the COVID-19 pandemic.

- Ethnicity
- Cultural factors
- Organisational role
- Work patterns
- Work experiences

**FEARS, CONCERNS, AND PERCEIVED RISK FACTORS**

**Big Idea:** There are likely to be individual level factors that healthcare workers are concerned about or feel put them at risk that relate to social, cultural, or personal factors, as well as organisational/institutional/structural factors.

- Individual fears and concerns
- Perceived risk factors
- Experiences of risk assessments
- Experiences of redeployment

**CHALLENGES RELATING TO ACCESSING INFORMATION OR ABILITY TO PROTECT ONESELF**

**Big Idea:** HCWs may have experienced factors in or outside the workplace that have impacted on their ability to access information or adequately protect themselves.

- Sources of information
- Experience in accessing information
- Experience of raising concerns

**SUPPORT AND FACILITATORS**

**Big Idea:** It is important to identify factors that have been helpful in supporting HCWs during the pandemic.

- Facilitators and coping mechanisms.
- Support at work
- Ability to raise concerns

**CULTURAL, ETHNIC, AND MIGRATION RELATED FACTORS**

**Big Idea:** Participants’ views can vary depending on their own background and social and cultural experiences.

- Influence of culture and ethnic factors on experiences.

**STIGMA, DISCRIMINATION, AND RACISM**

**Big Idea:** Stigma, discrimination, and racism may impact on people’s experiences during the pandemic, or put them at increased risk.

- Experiences of stigma, discrimination and racism inside and outside of work.

**PROTECTION AND PROMOTION**

**Big Idea:** What factors are important to think about going forward, both during this pandemic and future public health emergencies.

- Recommendation
- Vaccination and perception about vaccine

**CLOSING**

- Thank participant for their time
- Reminder of confidentiality
- Ask for potential to re-contact if there is there is a follow-up element in the research
- Ask for other potential interviewees.
